# Supplementary material for: A Topological Framework for the Computation of the HOMFLY Polynomial and Its Application to Proteins
Source: PLoS One. 2011 Apr 13;6(4):e18693. doi: 10.1371/journal.pone.0018693 (PMC3076383; doi:10.1371/journal.pone.0018693)
Supplement: Text S1 — Methods supporting information. This supplementary file details the computation of the intersection matrix and provides additional information on methods validation on tabulated knots and links and their application to protein structures. (PDF) [file pone.0018693.s001.pdf]

Supporting Information - Text S1

"A Topological Framework for the Computation of the HOMFLY Polynomial and its Application to Proteins"

Methods supporting information

Computation of the intersection matrix

Our intersection matrix computation considers the parametric equation of the lines in the plane containing respectively the segments $a = (a_1, a_2)$ and $b = (b_1, b_2)$

$$\begin{aligned} r_a(k_a) &= a_1 + k_a * (a_2 - a_1) \\ r_b(k_b) &= b_1 + k_b * (b_2 - b_1) \end{aligned}$$

and determine their intersection in terms of the parameters $(\bar{k}_a, \bar{k}_b)$. If $0 < \bar{k}_a < 1$ and $0 < \bar{k}_b < 1$ then the segments share a common point and we determine the sign of the corresponding layout in 3D according to the definition of the intersection signs (equation (2) in the main text).
The points

$$\begin{aligned} X_A = A_1 + \bar{k}_a * (A_2 - A_1) \\ X_B = B_1 + \bar{k}_b * (B_2 - B_1) \end{aligned}$$

belong to the segments A and B and $x = \pi_z(X_A) = \pi_z(X_B)$ is the intersection point. If $z(X_A) > z(X_B)$ the intersection sign is $+1$, otherwise -1. The case $z(X_A) = z(X_B)$ is not allowed since double points are not allowed.
Given two set of points $P = \{P_1, \ldots, P_n\}$ and $Q = \{Q_1, \ldots Q_m\}$ their intersection matrix $I = I(P, Q)$ is defined as the $(n-1) \times (m-1)$ matrix whose entries $I_{i,j}$ are equal to the intersection sign of the segments $P_i P_{i+1}$ and $Q_j Q_{j+1}$. Therefore $I(P) = I(P, P)$ is the standard antisymmetric intersection matrix.

Validation on tabulated knots and links

Three dimensional coordinates of knots up to 10 crossings (250 items) and of two (91), three (35) and four (4) components links were retrieved from the Knot Server (http://www.colab.sfu.ca/KnotPlot/KnotServer). Since the processing of symmetric structures could potentially give raise to numerical issues due to superimposed edges, we slightly perturbed the first two coordinates while preserving the topology.
Notice that for any tabulated knot or link the HOMFLY polynomial is not unique, due to two different sources of variation. First, chirality is not distinguished in the tabulation and this is related to the substitution $l = -l^{-1}$ in the HOMFLY polynomial. Secondly, the HOMFLY polynomial is sensitive to orientation and given an n component link, there are 2^n possible orientations. Since a global orientation change does not affect the polynomial, there are in principle 2^{n-1} possible different polynomials.
Generally, orientation is not considered in polynomial repositories. Thus, to obtain a complete polynomials repository and to compare our results with the HOMFLY polynomials provided by the Mathematica package KnotTheory (http://katlas.org) we considered mirror images and all possible orientations.

Application to protein structures

PDB entries (64046 structures) were fetched from the Protein Data Bank, version of November 8, 2010. Each structure was preprocessed as follows. Three dimensional coordinates of the protein backbone were retrieved and the presence of gaps was treated as chain terminator, globally giving raise to 210088 chain parts. Parts with less than 7 points were discarded since certainly trivial. A distances control (with a cutoff set to 5Å) on the protein backbone was performed in order to discard proteins with structural gaps.
Each structure was closed according to the center method referred in [1]. The terminals were placed on a

sphere having twice the radius of the enclosing sphere and artificially entangled structures were discarded (less than 3%). Finally, points were rotated via Principal Component Analysis projected along the direction given by the third component.

References

[1] Lua RC, Grosberg AY (2006) Statistics of knots, geometry of conformations, and evolution of proteins. PLoS Comput Biol 2: e45.
