# Supplementary material for: A Topological Framework for the Computation of the HOMFLY Polynomial and Its Application to Proteins
Source: PLoS One. 2011 Apr 13;6(4):e18693. doi: 10.1371/journal.pone.0018693 (PMC3076383; doi:10.1371/journal.pone.0018693)
Supplement: Text S2 — Generalized Reidemeister Moves. This supplementary file provides an illustrated description of a Generalized Reidemeister Move. (PDF) [file pone.0018693.s002.pdf]

Supporting Information - Text S2

“A Topological Framework for the Computation of the HOMFLY Polynomial and its Application to Proteins”

Generalized Reidemesteir Moves

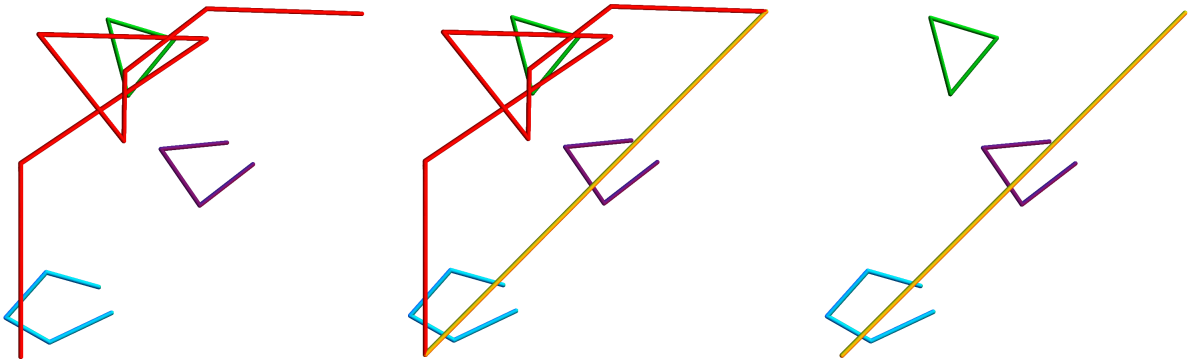

A B C

A four components polygonal link is shown. The move candidate $\mathcal{M}$ (in red) is ascending from left to right. Cyan, green and violet edges represent the polygonal link remainder, while the move contraction $\mathcal{M}^c$ is drawn in yellow.
Cyan and green components lay under $\mathcal{M}$ while the violet component does not intersect it (Figure A). On the other hand, cyan and violet components lay under $\mathcal{M}^c$ while the green component has no intersection with it (Figure B).
In the situation represented in the panels, $\mathcal{M}$ can be replaced by $\mathcal{M}^c$ because conditions TSC hold because $\mathcal{M}$ and $\mathcal{M}^c$ lay both on the same side of the link remainder and the move is trivial.
Generally, condition T is guaranteed by the triviality of $\mathcal{M}$, condition S implies that the move can be separated from the other components. Finally, condition C guarantees the concordance of $\mathcal{M}$ and $\mathcal{M}^c$ with respect to $\mathcal{L}$ and the feasibility of the move (Figure C).
In principle we could relax the conditions to include moves that allow heterogeneous signs provided that the components with different signs have the same sign or they do not overlap.
