# Supplementary material for: A Topological Framework for the Computation of the HOMFLY Polynomial and Its Application to Proteins
Source: PLoS One. 2011 Apr 13;6(4):e18693. doi: 10.1371/journal.pone.0018693 (PMC3076383; doi:10.1371/journal.pone.0018693)
Supplement: Text S3 — Mathematica code. Mathematica code for the computation of the HOMFLY polynomial of a polygonal link. An application example on the Rds3p protein (2k0a) is provided. (PDF) [file pone.0018693.s003.pdf]

Supporting Information - Text S3
"A Topological Framework for the Computation of the HOMFLY Polynomial and its Application to Proteins"

```
(****************************************************************************************)
(*This Mathematica notebook contains
 code written for the HPKnots Mathematica package*)
(*An application example is reported in the Example section*)
(****************************************************************************************)

BeginPackage["HPKnots`"]

(*Exported symbols*)

IntersectionMatrix::usage =
 "IntersectionMatrix[points3D] computes the intersection matrix of a set of 3D points."
MSR::usage = "MSR[points3D,ends,n] reduces a polygonal
   link (a set of 3D points along with a set of separators)
to its minimal structure. n is an iteration limit suitable to
   achieve a partial reduction (default is n=1000)"
HOMFLY::usage = "HOMFLY[points3D,skein,ends] computes the HOMFLY
   (Hoste, Ocneanu, Millett, Freyd, Lickorish, Yetter)
polynomial of a polygonal knot or link. The skein variable refers to the skein +1 or
   -1 sign. ends (the set of separators of link components) is an optional variable."
```

```
Rds3p = {{-9.225`, -24.265`, -3.881`}, {-9.927`, -20.804`, -5.217`},
    {-8.363`, -19.745`, -8.522`}, {-11.829`, -18.718`, -9.673`},
    {-14.645`, -16.69`, -8.082`}, {-12.639`, -15.926`, -4.953`},
    {-10.105`, -13.65`, -6.562`}, {-10.944`, -10.485`, -4.591`},
    {-9.794`, -8.744`, -7.783`}, {-6.918`, -7.069`, -5.969`},
    {-5.53`, -5.695`, -9.255`}, {-2.602`, -3.78`, -7.864`}, {0.183`, -1.847`, -9.551`},
    {2.885`, -4.313`, -8.45`}, {5.888`, -2.55`, -9.97`}, {9.243`, -1.803`, -8.385`},
    {8.738`, -0.064`, -4.985`}, {10.611`, 3.18`, -4.41`}, {11.295`, 5.116`, -1.224`},
    {7.896`, 6.783`, -0.902`}, {5.655`, 4.843`, 1.467`}, {2.08`, 4.025`, 0.496`},
    {-0.327`, 6.205`, 2.455`}, {-3.844`, 5.22`, 3.493`}, {-6.73`, 7.602`, 3.943`},
    {-8.458`, 7.184`, 7.327`}, {-11.35`, 5.602`, 5.43`}, {-9.095`, 2.701`, 4.378`},
    {-6.927`, 2.953`, 7.467`}, {-6.03`, -0.418`, 8.924`}, {-6.839`, -2.226`, 5.697`},
    {-4.147`, -4.439`, 4.245`}, {-3.813`, -3.418`, 0.558`}, {-3.285`, -7.028`, -0.484`},
    {-6.313`, -8.58`, 1.268`}, {-8.111`, -6.075`, 3.558`}, {-8.833`, -7.974`, 6.76`},
    {-7.781`, -5.63`, 9.587`}, {-5.192`, -8.138`, 10.762`}, {-1.704`, -7.831`, 12.309`},
    {0.677`, -5.797`, 10.09`}, {4.433`, -6.257`, 9.644`}, {6.025`, -3.85`, 7.17`},
    {5.175`, -0.497`, 5.582`}, {4.109`, -0.76`, 1.955`}, {6.391`, 0.907`, -0.574`},
    {4.79`, 2.337`, -3.692`}, {6.667`, 1.991`, -6.951`}, {8.639`, 5.017`, -8.113`},
    {6.269`, 5.211`, -11.09`}, {3.562`, 6.095`, -8.57`}, {5.844`, 8.216`, -6.391`},
    {6.833`, 10.513`, -9.219`}, {3.457`, 10.029`, -10.87`}, {1.21`, 12.406`, -8.894`},
    {-0.515`, 9.478`, -7.149`}, {1.95`, 9.438`, -4.322`}, {0.431`, 10.975`, -1.2`},
    {-3.138`, 9.989`, -2.093`}, {-4.475`, 6.822`, -0.474`}, {-3.233`, 3.718`, -2.235`},
    {-6.36`, 1.657`, -1.594`}, {-9.026`, 4.066`, -2.892`}, {-6.592`, 6.15`, -5.037`},
    {-8.561`, 9.333`, -4.439`}, {-8.454`, 10.761`, -0.926`}, {-5.014`, 11.769`, 0.344`},
    {-3.408`, 9.203`, 2.623`}, {-2.567`, 9.914`, 6.245`}, {-1.117`, 6.666`, 7.62`},
    {1.38`, 4.408`, 5.86`}, {-0.13`, 1.052`, 4.974`}, {1.374`, -2.112`, 6.374`},
    {1.422`, -5.496`, 4.683`}, {-0.38`, -7.886`, 7.007`}, {1.623`, -10.816`, 8.369`},
    {-0.589`, -13.271`, 6.473`}, {0.182`, -11.685`, 3.091`}, {3.861`, -11.537`, 4.033`},
    {3.835`, -15.214`, 4.998`}, {2.121`, -16.184`, 1.738`}, {4.457`, -13.91`, -0.174`},
    {1.984`, -11.572`, -1.825`}, {3.736`, -8.454`, -0.536`}, {6.948`, -9.469`, -2.309`},
    {5.24`, -8.682`, -5.614`}, {5.597`, -4.919`, -5.323`}, {3.595`, -1.68`, -5.442`},
    {0.376`, -2.235`, -3.422`}, {-1.635`, 0.475`, -5.174`}, {-5.167`, -0.729`, -5.775`},
    {-5.996`, -0.09`, -9.422`}, {-9.121`, -2.236`, -9.652`}, {-11.059`, 0.224`, -7.536`},
    {-12.951`, 2.511`, -9.857`}, {-15.981`, 2.561`, -7.606`}, {-17.523`, 0.44`, -4.816`},
    {-14.866`, 0.971`, -2.125`}, {-15.939`, -2.342`, -0.583`},
    {-18.588`, -4.953`, -1.356`}, {-21.574`, -3.397`, -3.148`},
    {-22.894`, -0.938`, -0.569`}, {-22.812`, 2.813`, -0.157`},
    {-25.728`, 2.739`, 2.29`}, {-26.629`, 6.43`, 2.613`}, {-23.026`, 7.647`, 2.636`},
    {-22.681`, 9.273`, -0.81`}, {-20.582`, 6.369`, -2.095`}, {-17.752`, 6.396`, 0.356`}};

Begin["`Private`"]
(*Implementation of the package*)
```

```
In[36]:= CheckSigns[A_] := Module[{}, Return[Min[A] * Max[A]]]
```

```
In[37]:= OrientedSign[points3D_, ij_] := Module[{orientedsign, vi, vj},
    vi = Join[points3D[[ij[[1, 2]], {1, 2}]] - points3D[[ij[[1, 1]], {1, 2}]], {0}];
    vj = Join[points3D[[ij[[2, 2]], {1, 2}]] - points3D[[ij[[2, 1]], {1, 2}]], {0}];
    orientedsign = Sign[Total[Cross[vj, vi]]];
    Return[orientedsign]
   ]
```

```
In[38]:= RemoveIndices[intersections_, indices_] := Module[{list, i},
    list = Delete[intersections, Table[{indices[[i]]}, {i, Length[indices]}]];
    Return[list]
   ]
```

```
In[39]:= CyclicShift[n_, shift_] := Module[{temp},
    temp = Mod[n + shift, 4] /. {0 → 4};
    Return[temp]
   ]
```

```
In[40]:= SingleIntersection[pointsij_, kind_] :=
  Module[{intersection = Switch[kind, "b", 0, _, {}], pointsij2D, v3D, v2D, det, ks,
    zs, sign, Xs, ksi, i, j, segment}, v3D = pointsij[[All, 2]] - pointsij[[All, 1]];
   v2D = v3D[[All, {1, 2}]];
   pointsij2D = pointsij[[All, All, {1, 2}]];
   det = Det[v2D];
   Switch[(det == 0),
    True,
    If[pointsij[[1, 1]] == pointsij[[1, 2]] || pointsij[[2, 1]] == pointsij[[2, 2]],
     Goto[Exit],
     If[MatrixRank[Transpose[Join[v2D, {pointsij2D[[2, 1]] - pointsij2D[[1, 1]]}]]] == 1,
       segment = pointsij2D[[1, 2]] - pointsij2D[[1, 1]];
       ksi = Table[(segment.(pointsij2D[[2, i]] - pointsij2D[[1, 1]])) /
          (segment.segment), {i, 2}];
       If[0 < ksi[[1]] < 1 || 0 < ksi[[2]] < 1, intersection = {};
        MessageDialog["SingleIntersection::Superimposed edges"];
        Goto[Exit]
       ];
      ];
    ],
    False,
    If[Length[Union[Flatten[pointsij, 1]]] < 4,
     Goto[Exit]
    ];
    ks = ({{v2D[[2, 2]], -v2D[[2, 1]]}, {v2D[[1, 2]], -v2D[[1, 1]]}} / det).
      (pointsij2D[[2, 1]] - pointsij2D[[1, 1]]);
    ks = Round[10^12 ks] / (10.^12);
    Switch[{(0 < ks[[1]] < 1 && 0 < ks[[2]] < 1), (kind == "b")},
     {True, True},
     intersection = 1;
     Goto[Exit],
     {False, True},
     intersection = 0;
     Goto[Exit],
     {True, False},
     zs = pointsij[[All, 1, 3]] + ks * v3D[[All, 3]];
     sign = Sign[zs[[1]] - zs[[2]]];
     Xs = Table[{}, {2}];
     If[sign == 1, i = 1; j = 2, i = 2; j = 1];
     Xs[[i]] = pointsij[[i, 1]] + ks[[i]] * v3D[[i]];
     Xs[[j]] = Xs[[i]];
     Xs[[j, 3]] = zs[[j]];
     intersection = Switch[kind,
       "sign", {sign},
       "x", {sign, Xs},
       "k", {sign, ks[[1]]},
       "lk", {sign, ks[[1]], Xs[[1, {1, 2}]], Xs},
       _, MessageDialog["SingleIntersection::Unproper kind option"];
      ],
     {False, False},
     Goto[Exit];
    ];
   ];
   Label[Exit];
   Return[intersection]]
```

In[41]:=
```
EdgeIntersections[points3D_, indices_, e1_] := Module[{edgeindices = indices[[e1 + {0, 1}]],
    n = Length[indices] - 1, intersections = {}, complement, temp, i},
   If[e1 > n,
    Goto[Exit],
    complement = DeleteCases[Table[indices[[{i, i + 1}]], {i, n}], edgeindices];
    Do[
     temp =
      SingleIntersection[{points3D[[edgeindices]], points3D[[complement[[i]]]]}, "x"];
     If[temp ≠ {},
      intersections = Join[intersections, {{complement[[i, 1]], temp}}];],
     {i, n - 1}
    ];
   ];
   Return[intersections];
   Label[Exit];
   MessageDialog["EdgeIntersections::edgeindex>maxindex."]];
```

In[42]:=
```
IntersectionMatrix[points3D_] :=
 Module[{nedge = Length[points3D] - 1, M, lintersections, n, intersecting, i, j},
  M = Table[0, {nedge}, {nedge}];
  lintersections = Table[EdgeIntersections[
     points3D[[Range[j + 1]]], Range[j + 1], j], {j, 2, nedge}];
  n = Map[Length, lintersections];
  intersecting = Flatten[Position[n, x_ /; x > 0], 1];
  n = n[[intersecting]];
  Do[
   Do[
    M[[intersecting[[i]] + 1, lintersections[[intersecting[[i]]]][[All, 1]][[j]]]] =
     lintersections[[intersecting[[i]]]][[All, 2, 1]][[j]], {j, n[[i]]}],
   {i, Length[intersecting]}];
  M -= Transpose[M];
  Return[M];
 ]
```

In[43]:=
```
GRM[points3D_, b_, ends_: {}, inputM_] :=
  Module[{points3Dout = points3D, endsout = ends, M = inputM,
    n = Length[points3D], ncomp = Length[ends] + 1, shift = 0, e, tempM, range,
    usubM, complement, complementsigns, newedge, nr, nrtemp, nrsigns, extends,
    comp, ecomponent, drop, Conditionfalse, Reduction, intervals, temp, k},
   If[b ≥ (n - 1) || MemberQ[ends, b],
    Goto[Exit];
   ];
   extends = Join[{0}, ends, {n}];
   intervals = Table[Range[extends[[k]] + 1, extends[[k + 1]] - 1], {k, ncomp}];
   comp = Position[extends, x_ /; x ≥ b][[1, 1]] - 1;
   ecomponent = extends[[comp + 1]];
   tempM = M;
   e = b;
   While[(e < ecomponent - 1),
    e++;
    range = Range[b, e];
    usubM = UpperTriangularize[M[[range, range]]];
    If[(CheckSigns[usubM] ≠ -1) == False,
     Goto[Conditionfalse];
    ];
    complement = Table[M[[range, intervals[[k]]]], {k, ncomp}];
    complement[[comp]] = complement[[comp]][[All,
       Complement[intervals[[comp]], Range[b, e]] - If[comp > 0, extends[[comp]], 0]]];
    complementsigns = Union[Flatten[complement]];
    temp = CheckSigns[complementsigns];
    If[(temp ≠ -1) == False,
     Goto[Conditionfalse];
    ];
    temp = EdgeIntersections[points3D, Drop[Range[n], {b + 1, e}], b];
    newedge = Transpose[{temp[[All, 1]], temp[[All, 2, 1]]}];
    nr = Table[0, {n - 1}];
    nr[[newedge[[All, 1]]]] = newedge[[All, 2]];
    nrtemp = nr;
    nrtemp[[{b, e}]] = 0;
    nrsigns = Union[Flatten[nrtemp]];
    If[nrsigns == {}, Goto[Reduction];];
    If[(CheckSigns[{complementsigns, nrsigns}] ≠ -1) == False,
     Goto[Conditionfalse],
     Label[Reduction];
     tempM = M;
     tempM[[b]] = nr;
     tempM[[All, b]] = -nr;
     tempM = Drop[tempM, {b + 1, e}, {b + 1, e}];
    ];
   ];
   shift = 1;
   Label[Conditionfalse];
   drop = shift + Min[e - 1, ecomponent - 2];
   If[b + 1 ≤ drop, points3Dout = Drop[points3Dout, {b + 1, drop}];
    endsout[[Range[comp, ncomp - 1]]] -= drop - b;
    M = tempM;];
   Label[Exit];
   Return[{points3Dout, endsout, M}];];
```

In[44]:=
```
MSR[points3D_, ends_, n_: 1000] :=
  Module[{points3Dout = points3D, endsout = ends, M, b, l, k, p, i},
   M = IntersectionMatrix[points3D];
   l := Length[points3Dout];
   k = Length[ends] + 1;
   Do[
    If[l ≤ 2 * k,
     Goto[Exit]
    ];
    p = Length[points3Dout];
    Do[
     {points3Dout, endsout, M} = GRM[points3Dout, b, endsout, M],
     {b, l - 1}
    ];
    If[p == l, Goto[Exit]],
    {i, n}];
   Label[Exit];
   Return[{points3Dout, endsout}];
  ];
```

In[45]:=
```
EvaluateVertex[points3D_, ends_, ij_, quadrilateral_, k_] :=
 Module[{intersections, eval, vertices, todel, j},
  vertices = {k, Mod[k, 4] + 1};
  intersections = Table[SingleIntersection[
     {{quadrilateral[[vertices[[1]]]], quadrilateral[[vertices[[2]]]]},
      {points3D[[j]], points3D[[j + 1]]}}, "b"], {j, Length[points3D] - 1}];
  todel = Join[ij[[All, 1]], ends];
  intersections = RemoveIndices[intersections, todel];
  eval = (Union[intersections] ≠ {0});
  Return[eval /. {True → 1, False → 0}]
 ]
```

In[46]:=
```
Xclean[points3D_, ends_, quadrilateral_, ij_, S_, toevaluate_] :=
 Module[{intersections = S, i, in, out, k, pos, neigh, j},
  Do[
   {in, out} = intersections[[toevaluate[[i]]]];
   Switch[{in, out},
    {1, 0}, k = {CyclicShift[toevaluate[[i]], -1]}; pos = {1}; neigh = {{k[[1]], 2}},
    {0, 1}, k = {toevaluate[[i]]}; pos = {2}; neigh = {{CyclicShift[k[[1]], +1], 1}},
    {1, 1}, k = {CyclicShift[toevaluate[[i]], -1], toevaluate[[i]]};
    pos = {1, 2}; neigh = {{k[[1]], 2}, {CyclicShift[k[[2]], +1], 1}}
   ];
   Do[
    intersections[[toevaluate[[i]], pos[[j]]]] =
     EvaluateVertex[points3D, ends, ij, quadrilateral, k[[j]]];
    intersections[[neigh[[j, 1]], neigh[[j, 2]]]] =
     intersections[[toevaluate[[i]], pos[[j]]]],
    {j, Length[k]}],
   {i, Length[toevaluate]}
  ];
  Return[intersections]]
```

In[47]:=
```
Quadrilateral[points3D_, ends_, pointsij_, ij_, Xs_, k_] :=
 Module[{ks, S, perm, vertices, i, distances, quad, flag, ones, tohalf},
  ks = Table[k, {4}];
  S = Table[{1, 1}, {4}];
  perm = {1, 3, 2, 4};
  distances = Table[0, {4}];
  vertices = pointsij[[perm]];
  Do[
   vertices[[i]] = Xs[[i]] + ks[[i]] * (vertices[[i]] - Xs[[i]]);
   distances[[i]] = Norm[Join[vertices[[i, {1, 2}]] - Xs[[i, {1, 2}]], {0}]],
   {i, Length[vertices]}
  ];
  quad = Transpose[{vertices, distances}];
  S = Xclean[points3D, ends, quad[[All, 1]], ij, S, Range[4]];
  If[Total[S, 2] == 0,
   Goto[Exit]
  ];
  flag = False;
  While[flag == False,
   ones = Union[Position[S, 1][[All, 1]]];
   tohalf = Position[quad[[All, 2]], Max[quad[[ones, 2]]]][[1, 1]];
   ks[[tohalf]] *= 0.5;
   quad[[tohalf, 1]] =
    Xs[[tohalf]] + ks[[tohalf]] * (pointsij[[perm]][[tohalf]] - Xs[[tohalf]]);
   quad[[tohalf, 2]] = Norm[Join[quad[[tohalf, 1, {1, 2}]] - Xs[[tohalf, {1, 2}]], {0}]];
   S[[tohalf]] = {1, 1};
   S = Xclean[points3D, ends, quad[[All, 1]], ij, S, {tohalf}];
   If[Total[S, 2] == 0,
    flag = True;
   ];
  ];
  Label[Exit];
  Return[quad[[perm, 1]]];
 ]
```

In[48]:=
```
RotateQuadrilateral[points3D_, ends_, quadrilateral_, ij_, Xs_, ialpha_] :=
  Module[{alpha = ialpha, R, S, perm, vertices, i, rquad, flag},
   perm = {1, 3, 2, 4};
   vertices = quadrilateral[[perm]];
   rquad = vertices;
   flag = False;
   While[flag == False,
    R = Transpose[RotationMatrix[alpha, {0, 0, 1}]];
    Do[
     rquad[[i]] = Flatten[Xs[[i]] + R.(vertices[[i]] - Xs[[i]]), 1],
     {i, Length[vertices]}
    ];
    S = Table[{1, 1}, {4}];
    S = Xclean[points3D, ends, rquad, ij, S, Range[4]];
    If[Total[S, 2] == 0,
     flag = True,
     alpha *= 0.5;
    ];
   ];
   Return[rquad[[perm]]]
  ];
```

In[49]:=
```
Triangles[points3D_, ends_, vertices_, quad_, rquad_, ij_] :=
 Module[{intersections, i, j, k, triangles, extendedij, n},
  intersections = Table[{}, {4}];
  extendedij = Sort[Flatten[Table[ij, {2}]]];
  n = Length[points3D] - 1;
  triangles = Transpose[{vertices, quad, rquad}];
  Do[intersections[[i]] = Table[RemoveIndices[
      Table[SingleIntersection[{{triangles[[i, j]], triangles[[i, Mod[j, 3] + 1]]},
         {points3D[[k]], points3D[[k + 1]]}}, "k"], {k, n}],
      Join[{extendedij[[i]]}, ends]], {j, Length[triangles[[i]]]}],
   {i, 4}
  ];
  Return[intersections]]
```

In[50]:=
```
CheckTriangles[triangles_] :=
 Module[{checklist, i, triangle, toedit, intposition, signcheck, ordercheck},
  checklist = Table[1, {4}];
  Do[
   triangle = triangles[[i]];
   toedit = Flatten[Position[triangle[[3]], x_ /; x ≠ {}], 1];
   triangle[[3, toedit, 2]] = 1 - triangle[[3, toedit, 2]];
   intposition = Table[Flatten[Position[triangle[[i]], x_ /; x ≠ {}], 1],
     {i, Length[triangle]}];
   If[intposition[[2]] == {} && (intposition[[1]] == intposition[[3]]),
    signcheck = (triangle[[3]][[toedit]][[All, 1]] == triangle[[1]][[toedit]][[All, 1]]);
    ordercheck = (Ordering[triangle[[3]][[toedit]][[All, 2]]] ==
       Ordering[triangle[[1]][[toedit]][[All, 2]]]);
    If[signcheck && ordercheck, checklist[[i]] = 0;
    ];
   ],
   {i, 4}
  ];
  Return[checklist];]
```

In[51]:=
```
AuxiliaryPoints[points3D_, ends_, orientedsigns_: {}] :=
  Module[{uM, nover, nunder, lengthLsw, savedLsw, moves, candidate, e1, e2,
    ij, pointsij, skeinsigns, v, w, Xij, Xs, quad, rquad, temp, alpha, flag,
    triangles, checked, Xsw, ksw, kz, Lsw, endsLsw, points3Dout, endsout, n, i},
   uM = UpperTriangularize[IntersectionMatrix[points3D]];
   uM[[ends]] *= 0;
   uM[[All, ends]] *= 0;
   moves = Position[uM, -1];
   {nover, nunder} = Map[Length, {Position[uM, 1], moves}];
   If[nover == 0 || nunder == 0,
    MessageDialog["AuxiliaryPoints:: no novercross or nundercross left"];
    Goto[Exit];
   ];
   n = Length[points3D];
   lengthLsw = Table[0, {nunder}];
   savedLsw = lengthLsw;
   Do[
    {e1, e2} = moves[[candidate]];
    ij = {e1 + {0, 1}, e2 + {0, 1}};
    pointsij = points3D[[Flatten[ij]]];
    skeinsigns = Join[orientedsigns, {OrientedSign[points3D, ij]}];
    v = Join[pointsij[[2, {1, 2}]] - pointsij[[1, {1, 2}]], {0}];
    w = Join[pointsij[[4, {1, 2}]] - pointsij[[3, {1, 2}]], {0}];
    Xij = SingleIntersection[
       {{pointsij[[1]], pointsij[[2]]}, {pointsij[[3]], pointsij[[4]]}}, "lk"][[4]];
    Xs = Join[Xij, Xij];
    quad = Quadrilateral[points3D, ends, pointsij, ij, Xs, 0.8];
    temp = VectorAngle[v, w];
    alpha = Min[(0.99 * temp), (0.99 * (Pi - temp)), Pi / 8.];
    alpha = alpha * Sign[Cross[v, w][[3]]];
    flag = False;
    While[flag == False,
     rquad = RotateQuadrilateral[points3D, ends, quad, ij, Xs, alpha];
     triangles = Triangles[points3D, ends, pointsij, quad, rquad, ij[[All, 1]]];
     checked = CheckTriangles[triangles];
     If[Total[checked] ≠ 0, alpha *= 0.5,
      flag = True
     ];
    ];
    ksw = 0.9;
    kz = 1;
    flag = False;
    While[flag == False, kz *= 2;
     Xsw = Xij[[1]] + kz * (Xij[[2]] - Xij[[1]]);
     Xsw = rquad[[1]] + ksw * (Xsw - rquad[[1]]);
     If[SingleIntersection[{{rquad[[2]], Xsw}, {rquad[[4]], rquad[[3]]}}, "k"][[1]] ==
        -1 && kz < 2^50,
      ksw += (1 - ksw) / 2, flag = True;
     ];
    ];
    Lsw = Join[points3D[[Range[e1]]],
      {rquad[[1]], Xsw, rquad[[2]]}, points3D[[Range[e1 + 1, n]]]];
    endsLsw = ends + Table[If[ends[[i]] > e1, 3, 0], {i, Length[ends]}];
    {points3Dout, endsout} = MSR[Lsw, endsLsw];
    lengthLsw[[candidate]] = Length[points3Dout];
    savedLsw[[candidate]] = {rquad, Xsw, e1, e2, skeinsigns}, {candidate, nunder}];
   Return[savedLsw[[Ordering[lengthLsw][[1]]]]];
   Label[Exit];
   Return[{}]
  ];
```

In[52]:=
```
LSwitch[points3D_, ends_, rquadrilateral_, Xsw_, e1_] :=
 Module[{n = Length[points3D], nends = Length[ends], Lsw, Lswends, shift, i},
  Lsw = Join[points3D[[Range[e1]]],
    {rquadrilateral[[1]], Xsw, rquadrilateral[[2]]}, points3D[[Range[e1 + 1, n]]]];
  shift = Table[If[ends[[i]] > e1, 3, 0], {i, nends}];
  Lswends = ends + shift;
  Return[{Lsw, Lswends}]]
```

In[53]:=
```
LZero[points3D_, ends_, rquadrilateral_, e1_, e2_] :=
 Module[{bpoints3D = points3D, bends = ends, temp, icomp,
   jcomp, samecomp, Lzero, Lzeroends, endsshift, i, n, nends},
  nends = Length[bends];
  n = Length[bpoints3D];
  temp = Sort[Join[bends, {e1}, {e2}]];
  icomp = Position[temp, e1][[1, 1]];
  jcomp = Position[temp, e2][[1, 1]] - 1;
  samecomp = (Abs[icomp - jcomp] == 0);
  If[samecomp,
   Lzero = Join[bpoints3D[[Range[e1]]], {rquadrilateral[[1]], rquadrilateral[[4]]},
     bpoints3D[[Range[e2 + 1, bends[[icomp]]]]], {rquadrilateral[[2]]},
     bpoints3D[[Range[e1 + 1, e2]]], {rquadrilateral[[3]], rquadrilateral[[2]]},
     If[icomp < nends, bpoints3D[[Range[bends[[icomp]] + 1, n]]], {}]];
   Lzeroends = Flatten[Join[{Table[bends[[i]], {i, icomp - 1}],
       bends[[icomp]] - e2 + e1 + 2, Table[bends[[i]] + 5, {i, icomp, nends}]}], 2],
   If[bends[[jcomp - 1]] + 1 == bends[[jcomp]],
    bpoints3D = Delete[bpoints3D, {bends[[jcomp]]}];
    bends[[Range[jcomp, nends]]] -= 1;
   ];
   Lzero = Join[bpoints3D[[Range[1, e1]]], {rquadrilateral[[1]], rquadrilateral[[4]]},
     bpoints3D[[Range[e2 + 1, bends[[jcomp]]]]],
     bpoints3D[[Range[bends[[jcomp - 1]] + 1, e2]]], {rquadrilateral[[3]]},
     {rquadrilateral[[2]]}, bpoints3D[[Range[e1 + 1, bends[[icomp]]]]],
     bpoints3D[[Complement[Range[bends[[icomp]] + 1, Last[bends]],
        Range[bends[[jcomp - 1]] + 1, bends[[jcomp]]]]]]];
   endsshift = 4 + (bends[[jcomp]] - bends[[jcomp - 1]]);
   Lzeroends =
    Flatten[Join[{Table[bends[[i]], {i, icomp - 1}], Table[bends[[i]] + endsshift,
        {i, icomp, jcomp - 1}]}, Table[bends[[i]] + 4, {i, jcomp + 1, nends}]]]];
  Return[{Lzero, Lzeroends}]
 ]
```

In[54]:=
```
SkeinIterator[points3D_, ends_, orientedsigns_: {}] :=
  Module[{leaves, innerv, signs, nalive, descendance, dsigns, newd, temp, ncomp,
    rquadrilateral, Xsw, e1, e2, Lsw, Lswends, Lzero, Lzeroends, points3Dout,
    endsout, dM, toleaves, up, down, i, j, k, dpoints, dextends, n}, Clear[tree];
   tree[{1}] = 0;
   ncomp = Length[ends] + 1;
   If[Length[points3D] == 2 * (ncomp),
    tree[{1}] = {points3D, ends, {}};
    leaves = {};
    Goto[Exit]
   ];
   innerv = {{1}};
   leaves = {{}};
   signs = orientedsigns;
   tree[innerv[[1]]] = {points3D, ends, {}};
   While[innerv ≠ {},
    nalive = Length[innerv];
    descendance = Flatten[Table[Table[Join[innerv[[i]], {j}], {j, 2}], {i, nalive}], 1];
    Do[
     dpoints = tree[innerv[[k]]][[1]];
     n = Length[dpoints];
     dextends = Join[tree[innerv[[k]]][[2]], {n}];
     dsigns = tree[innerv[[k]]][[3]];
     temp = AuxiliaryPoints[dpoints, Most[dextends], dsigns];
     If[temp == {},
      MessageDialog["SkeinIterator::no -1 left"];
      Goto[Exit]
     ];
     {rquadrilateral, Xsw, e1, e2, signs} = temp;
     newd = Join[innerv[[k]], {1}];
     {Lsw, Lswends} = LSwitch[dpoints, dextends, rquadrilateral, Xsw, e1];
     {points3Dout, endsout} = MSR[Lsw, Most[Lswends]];
     tree[newd] = {points3Dout, endsout, signs};
     newd = Join[innerv[[k]], {2}];
     {Lzero, Lzeroends} = LZero[dpoints, dextends, rquadrilateral, e1, e2];
     {points3Dout, endsout} = MSR[Lzero, Most[Lzeroends]];
     tree[newd] = {points3Dout, endsout, signs}, {k, nalive}];
    dM = Table[{}, {Length[descendance]}];
    Do[
     dM[[i]] = UpperTriangularize[IntersectionMatrix[tree[descendance[[i]]][[1]]]];
     dM[[i]][[tree[descendance[[i]]][[2]]]] *= 0;
     dM[[i]][[All, tree[descendance[[i]]][[2]]]] *= 0,
     {i, Length[descendance]}
    ];
    toleaves =
     Table[(Count[dM[[i]], -1, 2] == 0) || (Count[dM[[i]], 1, 2] == 0), {i, Length[dM]}];
    up = Flatten[Position[toleaves, False], 1];
    down = Flatten[Position[toleaves, True], 1];
    innerv = descendance[[up]];
    leaves = Join[leaves, {descendance[[down]]}];
   ];
   leaves = Rest[leaves];
   Label[Exit];
   Return[leaves]
  ];
```

In[55]:=
```
Correspondence[index_, sign_, l_, m_, skein_] := Module[{p = index * sign, correspondence},
  correspondence = Switch[p,
    1, -skein * l^(-2),
    2, -skein * l^(-1) * m,
    -1, -skein * l^2,
    -2, -m * l];
  Return[correspondence]
 ]
```

In[56]:=
```
Contribute[ancestor_, signs_, l_, m_, skein_] := Module[{n = Length[ancestor], i},
  Return[Product[Correspondence[ancestor[[i]], signs[[i]], l, m, skein], {i, n}]]
 ]
```

In[57]:=
```
HOMFLYComputation[leaves_, skein_, l_: l, m_: m] :=
  Module[{polynomial, nends, leavesv, n, nendsv, componentscontribute, ancestors,
    ancestorscontribute, signs, exponent, coeff, ncoeff, i}, polynomial = Infinity;
   If[(leaves == {}),
    nends = Length[tree[{1}][[2]]];
    polynomial = (m^(-1) * (-skein * l - l^(-1)))^nends, leavesv = Flatten[leaves, 1];
    n = Length[leavesv];
    nendsv = Map[Length, Table[tree[leavesv[[i]]][[2]], {i, n}]];
    componentscontribute = Table[(m^(-1) (-skein * l - l^(-1)))^(nendsv[[i]]), {i, n}];
    ancestors = Table[Rest[leavesv[[i]]], {i, n}];
    signs = Table[Flatten[tree[leavesv[[i]]][[3]]], {i, n}];
    ancestorscontribute =
     Table[Contribute[ancestors[[i]], signs[[i]], l, m, skein], {i, n}];
    polynomial = Simplify[Total[componentscontribute * ancestorscontribute]];
    exponent = Exponent[polynomial, m^-1];
    coeff = CoefficientList[polynomial * m^exponent, m];
    ncoeff = Length[coeff];
    polynomial = Total[coeff * Table[m^(-exponent + (i - 1)), {i, ncoeff}]];
   ];
   Return[polynomial]
  ];
```

In[58]:=
```
HOMFLY[points3D_, skein_, ends_: {}] :=
 Module[{polynomial = Infinity, points3Dout, endsout, leaves},
  Print["Input #points: ", Length[points3D]];
  Print["...Reducing structure..."];
  {points3Dout, endsout} = MSR[points3D, ends];
  Print["MSR completed. #points:", Length[points3Dout], " S:", endsout];
  leaves = SkeinIterator[points3Dout, endsout, {}];
  Print["...Computing Polynomial..."];
  polynomial = HOMFLYComputation[leaves, skein, l, m];
  Print["HOMFLY Polynomial (skein sign ", skein, "): ", polynomial];
  Return[{polynomial, points3Dout}]
 ]

End[]

EndPackage[]
```

Application to the Rds3p protein

In[35]:=
```
Import["http://www.rcsb.org/pdb/download/downloadFile.do?fileFormat=pdb&compression=NO&
   structureId=2k0a", "PDB", ImageSize → Small]
```

Out[35]=

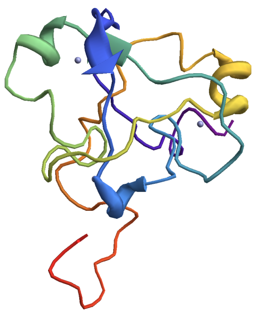

In[65]:=
```
<< HPKnots`
```

```
Loading HPKnots, version of November 20, 2010
```

In[66]:=
```
{points, ends} = MSR[Rds3p, {}]
```

Out[66]=
```
{{{-9.225, -24.265, -3.881}, {9.243, -1.803, -8.385},
  {-0.327, 6.205, 2.455}, {0.677, -5.797, 10.09}, {6.667, 1.991, -6.951},
  {-2.567, 9.914, 6.245}, {1.374, -2.112, 6.374}, {-17.752, 6.396, 0.356}}, {}}
```

In[67]:=
```
IntersectionMatrix[points] // MatrixForm
```

Out[67]//MatrixForm=

0	0	0	0	0	0	0
0	0	0	1	0	0	0
0	0	0	0	0	-1	1
0	-1	0	0	0	0	0
0	0	0	0	0	0	0
0	0	1	0	0	0	0
0	0	-1	0	0	0	0

In[68]:=
```
HOMFLY[Rds3p, -1]
```

```
Input ♯points: 109

...Reducing structure...

MSR completed. ♯points:8 S:{}

...Computing Polynomial...
```

HOMFLY Polynomial (skein sign -1): $2 l^2 - l^4 + l^2 m^2$

Out[68]=
$\{2 l^2 - l^4 + l^2 m^2$, {{-9.225, -24.265, -3.881}, {9.243, -1.803, -8.385},
{-0.327, 6.205, 2.455}, {0.677, -5.797, 10.09}, {6.667, 1.991, -6.951},
{-2.567, 9.914, 6.245}, {1.374, -2.112, 6.374}, {-17.752, 6.396, 0.356}}$\}$
