# Supplementary material for: A Topological Framework for the Computation of the HOMFLY Polynomial and Its Application to Proteins
Source: PLoS One. 2011 Apr 13;6(4):e18693. doi: 10.1371/journal.pone.0018693 (PMC3076383; doi:10.1371/journal.pone.0018693)
Supplement: Table S1 — Table of knotted PDB entries. This supplementary table provides PDB ID and part details for each database entry that revealed a knotted structure. Entries are conveniently grouped by knot type. (PDF) [file pone.0018693.s004.pdf]

Supporting Information - Table S1

"A Topological Framework for the Computation of the HOMFLY Polynomial and its Application to Proteins"

Table of knotted PDB entries

Knot Type	PDB Entry
3_1	1AJC (1-A), 1AZM (1-A), 1BZM (1-A), 1CRM (1-A), 1CZM (1-A), 1DMX (1-A, 2-B), 1DMY (1-A, 2-B), 1EOU (1-A), 1FUG (2-B), 1GZ0 (1-A, 2-B, 3-C, 4-D, 5-E, 6-F, 7-G, 8-H), 1HCB (1-A), 1HUG (1-A), 1HUH (1-A), 1IPA (2-A), 1J85 (1-A), 1J9W (1-A, 2-B), 1JS1 (1-X, 2-Y, 3-Z), 1JV0 (1-A, 2-B), 1K3R (1-A, 2-B), 1KEQ (1-A, 2-B), 1KOP (1-A, 2-B), 1KOQ (1-A, 2-B), 1MXI (1-A), 1NS5 (1-A, 2-B), 1NXZ (1-A, 2-B), 1O6D (1-A), 1OKL (1-A), 1OSN (1-A), 1P73 (4-B), 1P75 (1-A, 4-C, 6-D), 1P7C (5-B), 1P7L (1-A, 2-B, 3-C, 4-D), 1P9P (1-A), 1S1H (16-I), 1TO0 (2-A, 4-B, 6-C, 8-D, 10-E, 12-F, 14-G, 16-H), 1UAJ (1-A), 1UAL (1-A), 1UAM (1-A), 1URT (1-A), 1V2X (1-A), 1V6Z (1-A), 1V9E (1-A, 2-B), 1V9I (1-C), 1VH0 (1-A, 2-B, 5-D, 6-E, 8-F), 1VHY (2-A, 5-B), 1X7O (1-A, 4-B), 1X7P (1-A, 4-B), 1Y7W (1-A, 2-B), 1YO0 (1-A), 1YO1 (1-A), 1ZJR (1-A), 2CAB (1-A), 2CX8 (1-A, 2-B), 2EGV (1-A, 2-B), 2EGW (1-A, 2-B), 2FG6 (1-C, 2-D, 3-E, 4-X, 5-Y), 2FG7 (1-C, 2-D, 3-E, 4-X, 5-Y, 6-Z), 2FOY (1-A, 2-B), 2FW4 (1-A, 2-B), 2G7M (3-E, 4-X, 5-Y, 6-Z), 2HA8 (1-A, 2-B), 2HFX (1-A), 2HKK (1-A) 2I6D (1-A), 2IT4 (1-A, 2-B), 2IUC (1-A, 3-B), 2NMX (1-A, 2-B), 2NN1 (1-A, 2-B), 2NN7 (1-A, 2-B), 2NXT (1-A), 2OBV (1-A), 2OSF (1-A), 2OSM (1-A), 2P02 (1-A), 2QMM (1-A, 2-B), 2V3J (3-A), 2V3K (2-A), 2WD3 (1-A), 2X4I (1-A, 5-C, 8-D), 2X7T (1-A), 2X7U (1-A), 2YY8 (1-A, 2-B), 2Z0Y (1-A), 3BBD (1-A, 2-B), 3BBE (1-A, 2-B), 3CZV (1-A, 2-B), 3D0N (1-A, 2-B), 3DA2 (1-A, 2-B), 3DCM (1-X), 3E5Y (1-A, 3-B), 3GYQ (1-A, 2-B), 3HKN (1-A), 3HKQ (1-A), 3HKT (1-A), 3HKU (1-A), 3IEF (2-A, 4-B), 3ILK (1-A, 3-B), 3JXF (1-A, 2-B), 3JXG (5-C), 3JYW (10-E), 3KNU (2-A, 4-B, 6-C, 9-D), 3KTY (4-B, 5-C), 3MDZ (1-A)
3_1^*	2EFV (1-A), 2K0A (1-A), 2RH3 (1-A)
4_1	1QMG (1-A, 2-B, 3-C, 4-D), 1YRL (1-A, 3-B, 4-C, 7-D), 1YVE (1-I, 2-J, 3-K, 4-L) 2O9C (1-A), 3C2W (1-A, 3-B, 6-C, 8-D, 19-G, 21-H), 3FR8 (1-A, 2-B), 3G6O (1-A, 4-B), 3IBR (1-A, 4-B) 3LRB (1-A, 3-B), 3LRC (1-A, 3-B, 5-C, 7-D)
5_2^*	1XD3 (1-A, 3-C), 2ETL (2-A, 2-B)
6_1	3BJX (1-A, 2-B, 3-C, 4-D)
